# A Wake-Up Call: We Need Phage Therapy Now

**DOI:** 10.3390/v10120688

**Published:** 2018-12-05

**Authors:** Karin Moelling, Felix Broecker, Christian Willy

**Affiliations:** 1 Institute of Medical Microbiology, University of Zurich, 8006 Zurich, Switzerland; 2 Max Planck Institute for molecular Genetics, 14195 Berlin, Germany; 3 Department of Microbiology, Icahn School of Medicine at Mount Sinai, New York, NY 10029, USA; 4 Department Trauma & Orthopedic Surgery, Septic & Reconstructive Surgery, Research and Treatment Center for Complex Combat Injuries, Military Hospital Berlin, 10115 Berlin, Germany; christianwilly@bundeswehr.org

**Keywords:** phages, phage therapy, Helsinki declaration, regulation, magistral preparation

## Abstract

The rise of multidrug-resistant bacteria has resulted in an increased interest in phage therapy, which historically preceded antibiotic treatment against bacterial infections. To date, there have been no reports of serious adverse events caused by phages. They have been successfully used to cure human diseases in Eastern Europe for many decades. More recently, clinical trials and case reports for a variety of indications have shown promising results. However, major hurdles to the introduction of phage therapy in the Western world are the regulatory and legal frameworks. Present regulations may take a decade or longer to be fulfilled. It is of urgent need to speed up the availability of phage therapy.

## 1. Introduction

Phages are attracting increasing attention for therapeutic approaches against antibiotic-resistant bacterial infections [1]. They exert therapeutic effects by killing bacteria and have been applied successfully against various bacterial infections since their discovery about 100 years ago. However, no phage therapeutic approach has been approved for human use in the United States (US) or the European Union (EU). Serious adverse events by phage therapy have not been reported to date, which justifies their use against antibiotic-resistant bacterial infections [2]. Phages are biologicals and cannot fulfil the strict regulations for chemical antibacterial agents with respect to standardization and reproducibility. The Centers for Disease Control have warned about the present time as “post-antibiotic era” [3]. A group of commonly multidrug-resistant bacteria have been designated as ESKAPE, an acronym of *Enterococcus faecium*, *Staphylococcus aureus*, *Klebsiella pneumoniae*, *Acinetobacter baumannii*, *Pseudomonas aeruginosa*, and *Enterobacter* species [4]. Phage therapy could provide a promising adjunct to existing antibiotic treatments to combat multidrug-resistant infections.

## 2. History of Phage Therapy

Phages are the most abundant organisms on Earth. They amount to about 10^31^ particles on our planet and are distributed over all ecosystems such as the oceans and freshwater, the air up to the stratosphere, and surfaces inside and outside of the human body [5]. Depending on the environment, phages can be about 10- to 100-fold more abundant than their prokaryotic hosts [6]. In 2005, virologist Curtis Suttle illustrated their vast abundance by concentrating hundreds of liters of ocean water and presenting the phages in electron micrographs or by fluorescent DNA staining [7,8]. Earlier, in 1989, Norwegian scientists had described up to 250 million phages per milliliter of marine water using transmission electron microscopy, changing in numbers as a consequence of the seasons [9]. These numbers were unexpected and 10^3^–10^7^ times higher than those described before, which were based on counting plaque-forming units on various host bacteria. Recently, thousands of new phages have been identified in the oceans with genes influencing carbon, sulfur, phosphorous and nitrogen cycles, amongst others [10,11].

In 2017, the Institut Pasteur in Paris celebrated the centennial of the publication of a manuscript on “invisible antagonists” of bacteria isolated from patients with dysentery, published by Félix d’Herelle (1873–1949). The Franco-Canadian self-taught scientist with no university education was not highly respected by his academic fellow researchers. In 1917, he isolated the agents which he termed “bacteriophages” from human feces and used them to kill bacteria under laboratory conditions [12]. Two years earlier and independently of d’Herelle, the British bacteriologist Frederick Twort (1877–1950) discovered a “bacteriolytic agent” in the filtrate of bacterial cultures [13].

After finding that avian typhoid in chickens could be cured by phage cultures, d’Herelle’s findings were quickly applied to humans [12]. In 1921, patients at a children’s hospital in Paris were cured from toxic dysentery by oral phage application within one day. In Alexandria, Egypt, d’Herelle treated four patients suffering from bubonic plague by injecting phages directly into the infected lymph nodes [14]. All four patients recovered. This was a remarkable success and widely reported in French medical journals. During a cholera epidemic in India in 1927, d’Herelle treated patients with phages, reducing the mortality rate from 63% of untreated patients to 8% of phage treated patients.

**Figure 1:**
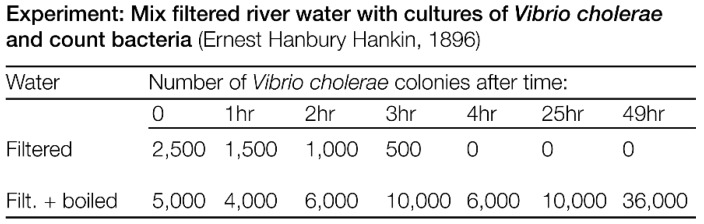
Effect of water from the Junma river on *Vibrio cholerae* bacteria (data from [15]). It is debated if the antibacterial effect was due to phages or other agents present in the river water [16].

In India, the English bacteriologist Ernest Hankin (1865–1939) had already detected the antibacterial activity of water from Indian rivers, without knowing about the agent [15]. While unboiled river water from the Junma river efficiently killed *Vibrio cholerae*, boiled water did not (Figure 1). It is debated if the antibacterial activity of the river water was due to phages or caused by a volatile compound. Abedon and colleagues [16] argued against phages as cause. However, since the exact experimental conditions remain unknown, some of the effects observed by Hankin could possibly have been due to phage activity. During the history of medicine animal dung was used to treat bacterial infections [17,18,19,20]. The cures were later on considered as myths. Yet, since feces contain up to 10^10^ phage particles per gram dry weight [21], some of the curing effects may have been due to phages.

The treatments performed by d’Herelle were not always easily reproducible by his colleagues [22]. He tried to overcome their criticism by combining different phages, demonstrating that cocktails increased the probability of combating diverse bacterial infections. Phage cocktails, combinations of phages, are still in use today and commercially available, for instance, in Russia [23]. This already indicated the high degree of specificity of phages towards their hosts. In 1923 d’Herelle, together with George Eliava (1892–1937), founded the Eliava Institute for Phage Therapy in the Republic of Georgia that still exists.

Phage therapy was massively implemented during the Winter War between the former Soviet Union and Finland (1939–1940), with 6,000 Soviet soldiers treated against open wounds with streptococcal or staphylococcal infections, which prevented amputations [24]. The wounds were topically treated, for example, with mixtures of streptococcal and staphylococcal phages or with pyophage (“PYO”) cocktail that contains phages to a wide variety of bacteria including streptococci, staphylococci, *Shigella*, *Salmonella*, *Escherichia coli*, *Pseudomonas aeruginosa* and *Proteus* [25]. Thereby, mortality due to gangrene was reduced to a third [24]. German companies such as Behring and Eli Lilly in the US produced phage preparations against streptococci, staphylococci and *Escherichia coli*. During World War II in Africa, the German army under general Erwin Rommel, as well as allied forces, applied phage therapy against dysentery [26].

In 1928, modern antibiotics were discovered and were first used on a large-scale during World War II [27]. The definitive abandonment of commercial phages in the 1980s was marked by the destruction of phage collections of the Pasteur Institutes in Paris and Lyon [28]—to the disappointment of many researchers today.

## 3. Fecal Microbiota Transplantation of the “Zurich Patient”– a Therapy against *Clostridium difficile* Infection Involving Phages

**Figure 2:**
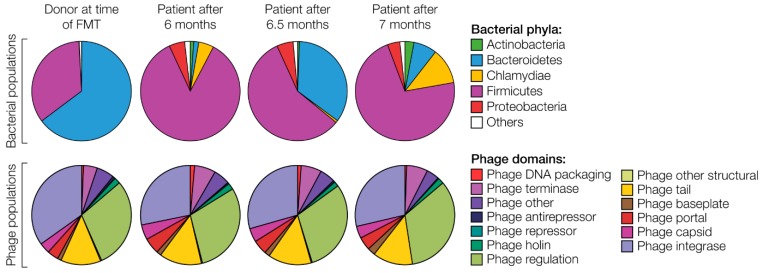
Dynamics of bacterial (top) and phage (bottom) populations in a *C. difficile* patient successfully treated by fecal microbiota transplantation (FMT). Data adapted from [30].

In 2008 a patient in Zurich came into the Institute of Microbiology, and asked for help in her case of chronic diarrhea caused by *C. difficile* infection resulting from antibiotic therapy for a jawbone infection. Against the original refusal by members of the medical faculty of the University Hospital of Zurich the patient insisted and in 2010 received fecal microbiota transplantation (FMT) from her sister as donor, under normal laboratory conditions (not a surgery unit) as one of the first patients in Central Europe to receive such a treatment. The patient was cured within a few days [29]. We have followed changes in the microbiome and virome for now eight years [29,30,31]. The patient recovered within days, even though the microbiota composition changed but remained distinct even after seven months. Interestingly, the intestinal phage population of the patient quickly became similar to that of the donor (Figure 2). Indeed, FMT could be regarded more as a phage therapy than a bacteriotherapy, as phages are by far the most abundant entities in human feces [32,33,34]. The patient’s bacterial population eventually became donor-like within four years as well [30,31].

In May of 2013, the US Food and Drug Administration stated that an investigational new drug administration was required to perform FMT, following the reporting of some initial case studies of this procedure [35]. This restriction was put into place because of theoretical safety concerns of transmission of diseases and the lack of standardization and testing. Only one month later, opposition from affected patients with recurrent *C. difficile* infection with no other therapeutic options and the convincing efficacy and safety data caused the FDA to reverse its stance and approved FMT for this indication [35]. FMT is by no means a new treatment – the first records date back to the China of the 4th century, where stool diluted with water and perhaps fermented, called “yellow soup”, was used against food poisoning and diarrhea [36]. The procedure is easy, cheap, has not been linked to any serious adverse events in over a century of applications, including these more well-documented recent cases, and shows an impressive cure rate of about 90% against recurrent *C. difficile* infection [37,38]. Consequently, FMT has gained acceptance within the medical community within a few years, and may also be effective against other indications such as inflammatory bowel disease (IBD), obesity, possibly even mental disorders, and others [39].

Analysis of the Zurich patient’s fecal virome revealed a relatively low complexity after recovery. Only about twenty different phage types were identified [31]. The relatively low abundance of phages in the cured patient may have been an indicator of a healthy microbiota. Indeed, inflammatory conditions associated with obesity and IBD lead to an expansion of phage populations [40,41,42,43]. In the case of the Zurich patient we identified a core phage population that was highly similar between donor and patient and therefore likely transmitted during FMT. In addition to these highly abundant core phages, individual phages of low abundance are likely present, but more difficult to identify. Similar to this analysis, viral populations in various sites of the oceans exhibited a core virome shared among samples from different sites, along with less abundant phages specific for certain environments [44].

Microbiota studies on the effects of FMT have largely focused on bacterial populations. However, it is likely that successful FMT is also attributable to the phages present in feces (the “phagebiota”), not to bacteria alone. Along this line, it has been demonstrated that FMT can cure *C. difficile* infections even when bacteria are removed by filtration, leaving phages as the only transmitted biological species [45]. Additionally, confirming the findings in the Zurich patient, it has been shown that transfer of phages during FMT correlates with success of treating *C. difficile* infections [46]. Thus, phages alone may be sufficient to exert therapeutic effects. This, however, needs to be confirmed with more patients. In the future, FMT may be replaced by microbiota-targeting therapies using defined combinations of microorganisms instead of complex and poorly defined donor stool specimens [47]. It can be envisioned that such combinations of microorganisms will benefit from including specific phages that can help establish a healthy microbiota in the patient. In the case of *C. difficile* infections, for example, it may also be useful to supplement microbiota-targeting therapies with a cocktail of *C. difficile*-specific phages [48]. The apparent importance of the phagebiota suggests that phage-based therapies may be efficient also against other intestinal diseases characterized by altered microbiota, such as IBD, type II diabetes (T2D), and obesity. Indeed, significant alterations in the phageomes of patients with IBD [43] and T2D [49] have been described, and phages adhering to intestinal mucus have been shown to provide protection against bacterial infections and regulate local inflammation [50,51]. These findings suggest that specifically manipulating phage populations may provide new avenues for curing these and related diseases.

Mouse studies have shown that obese mice feeding on the feces of co-caged lean mice can become lean [52]. Obese mice, on average, have less complex microbiota with reduced diversity and richness, perhaps because the rich nutritional milieu provided by a high caloric diet, causes overgrowth of a limited number of specialized bacteria [53,54,55]. Thus, obese mice may replenish their microbiota with the diverse microorganisms in the feces of lean mice. Low bacterial richness may also be linked to obesity in humans, which is apparently not easily reverted by patients [56]. It has been recently shown that a low diversity of intestinal bacteria predisposes to later weight gain, suggesting a causal role of microbiota in the development of obesity [57]. The role of the phageome, however, remains to be studied in human subjects.

## 4. Recent Developments in Phage Therapy

The fear of multidrug-resistant bacterial infections of hospitalized patients has led to recent efforts to investigate phage therapy by medical doctors, funding from the EU, and various companies. The Eliava institute in Tbilisi, Republic of Georgia, as well as hospitals in Novosibirsk, Russia and Wroclaw, Poland, have continuously published case reports on the positive effects of phage therapy and thereby helped to keep this approach alive [58,59,60,61,62]. Results from the first placebo-controlled, double-blind human clinical trial (Phase I/II) on therapeutic phages were published in 2009 [63]. Phages against *P. aeruginosa* infections in chronic otitis resulted in reduced bacterial counts and significantly improved symptoms compared to a placebo group, in the absence of treatment-related adverse events.

Most striking was the recent well-documented case of Tom Patterson who was infected with a multidrug-resistant *Acinetobacter baumannii* strain during a trip to Egypt. His wife, the physician Steffanie Strathdee, initiated a phage treatment. Patterson was transferred to California and remained in a coma for three months. The phage therapy was made possible through a combined effort by doctors, researchers, the US Navy and health authorities. The phages were isolates from sewage water and environmental samples. About 100 phages were screened for lytic activity against the patient’s *A. baumannii* strain and the most active phages were combined in cocktails of four. Three originated from the US Navy phage library at Texas A&M University, and one from AmpliPhi Biosciences, CA, and were applied through percutaneous catheters and intravenously, which was rather unusual but permitted under these life-threatening conditions. The treatment was well tolerated and the patient recovered [64]. Other nosocomial outbreaks of *A. baumannii* have been reported recently [65,66,67,68,69,70]. Phages may be able to contain such outbreaks and reduce mortality in the future.

Meanwhile, Strathdee wrote a book on how phage therapy saved her husband from a deadly superbug (to be published in 2019), and together with Robert Schooley became Codirector of University of California’s Center on Innovative Phage Applications and Therapeutics (IPATH) in San Diego, CA. Schooley, an infectious disease physician at the university, was the first author on the manuscript about Patterson’s cure [64]. Several compassionate trials on patients are forthcoming.

The Nestlé company started a large effort on phage therapy several years ago, described by the phage researcher Harald Brüssow. His group selected T4-like phages against *E. coli* induced diarrhea in children in Bangladesh. The orally applied phages were well-tolerated. However, the children did not have sufficient numbers of *E. coli* in their intestine to allow for amplification of the phages. Consequently, there was no significant therapeutic effect [71,72].

In 2013 the European Commission started a therapeutic trial for the treatment of burn wounds under the 7th Framework Program called PhagoBurn. The multicentered randomized clinical trial comprising eleven clinical EU partners from France, Belgium and Switzerland aimed at treating *P. aeruginosa* infected burn wounds with Good Manufacturing Practice (GMP) produced phages [73]. The topically applied phages successfully reduced the bacterial burden, but at slower pace than the standard of care treatment, sulfadiazine silver emulsion cream. The phages, however, caused fewer side effects than the standard of care treatment [73], highlighting the potential of phage therapy against open wounds. As part of the PhagoBurn trial, the French company Clean Cells was the first to achieve GMP-like production of phages.

In addition, efforts made in Germany by Phage4Cure, a joint project involving the Leibnitz Institut Deutsche Sammlung von Mikroorganismen und Zellkulturen (DSMZ) and the Fraunhofer Institute for Toxicology and Experimental Medicine in Braunschweig as well as the Charité Berlin with funding from the Ministry of Education and Research (BMBF) aim at identifying phages against *P. aeruginosa*, and test them in preclinical and clinical trials in the upcoming four years.

A significant reduction of a contamination with pan-resistant *P. aeruginosa* was achieved by surgeons of the German Military Hospital, Berlin, while treating a war-injured patient with a mixture of phages from the Eliava Institute, antibiotics and other detoxifying agents. The authors stress the importance of combining phages with antibiotics and even discuss antimicrobial peptides or photodynamic therapies in combination with phages [74]. Phages may increase the uptake of antibiotics into bacterial cells. Therefore, phage therapy is likely most effective when combined with antibiotics [74,75,76,77,78]. In this effort Jean-Paul Pirnay from the Queen Astrid Military Hospital in Brussels, Belgium, also participated, who is an advocate of phage therapy and published a proposal for guidelines on how phage therapy can be implemented today [1].

Another strong advocate of phage therapy, Elizabeth Kutter, is promoting phage treatment of diabetic foot ulcers that are often infected with MRSA strains or other multidrug resistant bacteria. The results of nine patients with MRSA infection and poor response to antibiotics that have been successfully treated topically with a staphylococcal phage have been published recently [79]. In addition, phage therapy shows promise against the second most frequently implicated bacterium in diabetic foot infections, *Klebsiella pneumoniae* [80]. In the US, about 100,000 foot amputations result from diabetic foot ulcers and osteomyelitis every year [81], many of which may be preventable by phage therapy.

In vitro studies performed in collaboration with the University Hospital of Zurich and the Eliava Institute on the PYO phage cocktail that is commercially available and registered in Russia identified a potential to cure urinary tract infections (UTIs) [82]. The laboratory results will be applied in a clinical trial to about 80 UTI patients who will be treated at the Tsulukidze National Center of Urology in Tbilisi, Republic of Georgia [83]. Phages will be prepared at the Eliava Institute. Pilot studies found that treatment of UTIs with the phage cocktails substantially decreased bacterial titers in six out of nine patients, with no adverse events reported [84].

The survey on phage therapy, as presented and discussed during the 2018 workshop Viruses of Microbes in Wroclaw, supported by the European Molecular Biology Organization (EMBO) and with over 500 participants, made it clear: phage therapy is not within close reach; it will likely take ten to fifteen years. Many of the participants had experienced desperate pleas and calls from patients with chronic infections facing an amputation as the only option. Others reported on family members who died of sepsis—without any explanation as to how this came about, such as hospital infections. Sepsis is a severe condition, however, it is not always life-threatening and therefore such patients do not comply with the rules of the Helsinki Declaration: “In the treatment of an individual patient, where proven interventions do not exist or other known interventions have been ineffective, the physician, after seeking expert advice, with informed consent from the patient or a legally authorized representative, may use an unproven intervention if in the physician’s judgement it offers hope of saving life, re-establishing health or alleviating suffering. This intervention should subsequently be made the object of research, designed to evaluate its safety and efficacy. In all cases, new information must be recorded and, where appropriate, made publicly available.” [85].

**Figure 3:**
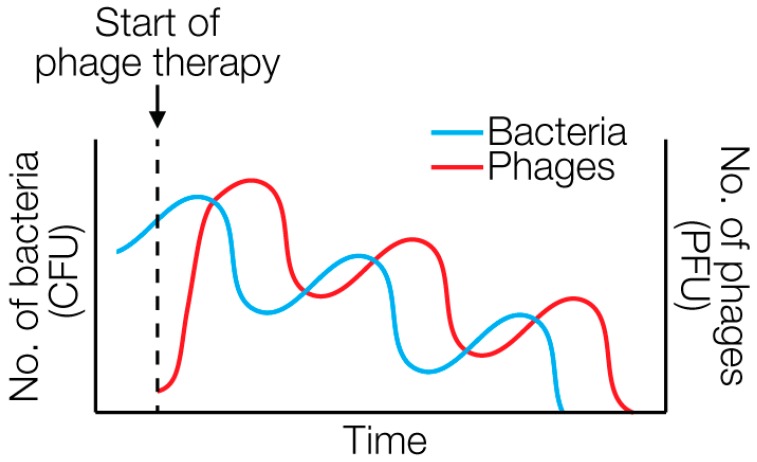
Population dynamics of bacteria and phages during phage therapy. The figure does not show actual data but represents typical growth curves observed experimentally. Phages are self-dosing (i.e., amplifying in the presence of the host bacterium) and self-limiting (i.e., cleared once the host bacterium is eliminated). PFU, plaque-forming units; CFU, colony-forming units.

As opposed to antibiotics, phages are self-dosing and self-limiting (Figure 3), and formation of resistance is typically lower [86,87,88]. Throughout more than one hundred years no serious adverse events caused by phage therapy have been reported [2,89]. However, there is still a need to develop or follow measures that can mitigate potential safety concerns. One of the challenges is that phage preparations will inevitably contain some amount of endotoxin or other harmful bacterial components. This is especially important if the phages are applied intravenously. Thus, before intravenous application, endotoxin levels need to be determined and the phage preparations diluted accordingly to meet the FDA-recommended limitation of 5 endotoxin units per kg of body weight per hour [64]. This issue can be overcome by using highly purified phage preparations [86]. Another safety concern is the potential rapid release of endotoxin due to bacterial lysis [90]. A strategy to avoid this is to genetically engineer non-replicating phages to inactivate the host bacteria without lysing them [91]. However, a recent study found that, although phages kill pathogenic *E. coli* faster than β-lactam antibiotics, phages released fewer endotoxin when directly compared [92].

Bacterial resistance to therapeutic phages has been observed. However, the potential for inducing resistance is considerably narrower than for antibiotics because of the restricted host range of most phages, as well as due to the fitness cost for the bacterium associated with altering surface receptors [82,83]. Since there is usually no cross-resistance with antibiotics, a combination of phages with antibiotics may be the best measure to preclude resistance formation [86].

The potential problem of introducing harmful genes such as virulence factors or those conferring antibiotic resistance can be overcome by using fully sequenced phages. Phage banks should only include well-characterized phages that do not carry potentially harmful genes.

Within the narrow time frame of approximately 36 h to treat septic patients, it is necessary for the physician to perform a “phagogram”, similar to an antibiogram, that will determine which phages the bacterial pathogens are susceptible to [1]. Of note, the results obtained from a phagogram are generally obtained within the same time frame as are antibiograms, within 18 h, using commercially available kits [1,93]. Selected phages can then be amplified and purified on-site within a time frame of 18 h [94].

## 5. The Magistral Approach

The costly and time-consuming requirements for the production of phages under current guidelines in the US and the EU are not easily fulfilled [1]. As a consequence, Belgium is currently implementing a pragmatic framework on phage therapy that centers on magistral preparation of individual therapeutic phages by pharmacies, whereby non-authorized ingredients (the phages) may be included, provided they have a certificate of analysis from a Belgian Approved Laboratory [1]. Although the final products will not fully comply with the European requirements for medicinal products for human use (Directive 2001/83/EC), such magistral phage preparations can be used to treat patients in Belgium. This approach provides an example of how to accelerate the implementation of phage therapy.

In contrast to conventional medicinal products approved under the present regulatory framework of the EU, which distributes responsibilities for safety between the prescriber, the pharmacist, the marketing authorization holder and regulatory authorities, a magistral formula prescription puts most of the liability on the prescribing physician and the pharmacist [95]. As a long-term solution to this problem, it has been suggested that novel EU regulations need to be implemented which allow for “Biological Master Files” (BMF), similar to procedures already existing for chemical drugs but not for biologicals such as phages. Similar to an Active Substance Master File (ASMF), the currently recognized term in the EU, a BMF for phages could cover manufacturing processes, quality control measures and compliance, as well as safety issues (including, for instance, endotoxin levels). The finished product could then be prepared as a magistral formula. In this scenario, liability would be shared more evenly between manufacturers, regulatory bodies, prescriber and pharmacist.

## 6. The Future of Phage Therapy

What is essential to accelerate the availability of phage therapy?

1. We need interim regulations by authorities with reduced stringency until the demands for present-day guidelines can be fulfilled, which may require many years.

2. We need well-characterized phages that are pure, sequenced and have a defined host specificity. Information on phage banks should become available to physicians. A phage bank with well-characterized phage stocks needs to be able to supply phages for fast amplification and treatment within 36 h—the time some septic patients spend in intensive care units before succumbing. Such a treatment would probably be in agreement with the Helsinki Declaration. However, physicians and patients need to be informed about phage therapy, informed consent is required and phages should be made available. For these phages, one or several authorized storage places are required. Characterization, purification, sequencing and storage of one phage can be achieved at a cost of about € 500. Storage under qualified conditions is available, for instance, in Bern, Switzerland. Large phage collections already exist in Brussels, Belgium, Tbilisi, Republic of Georgia, Novosibirsk, Russia, Braunschweig, Germany, Zurich, Switzerland, Helsinki, Finland, and Quebec City, Canada. They are, however, currently, not easily available.

Many phages are required, because multiple phage types may be needed to treat different strains of one bacterial species. Furthermore, several bacterial strains are often present in an infection. Phage therapy is a form of individualized or personalized medicine. Thus, therapeutic phages need to be tested for effectiveness against the patients’ pathogens (phagogram) and phage cocktails be individually prepared. This may not be attractive for industries, but is necessary.

3. The preparation of phages “in the spirit of GMP” should be performed with the help of magistral preparations by pharmacies as recommended in a concept paper by Pirnay and colleagues [1]. In the magistral approach, phage therapeutics are formulated on demand by selected authorized pharmacies.

4. Politicians need to be alerted to support such a combined effort, preferably at the European level. A fast interim solution is urgently needed and requires political decisions to be made.

**Figure 4:**
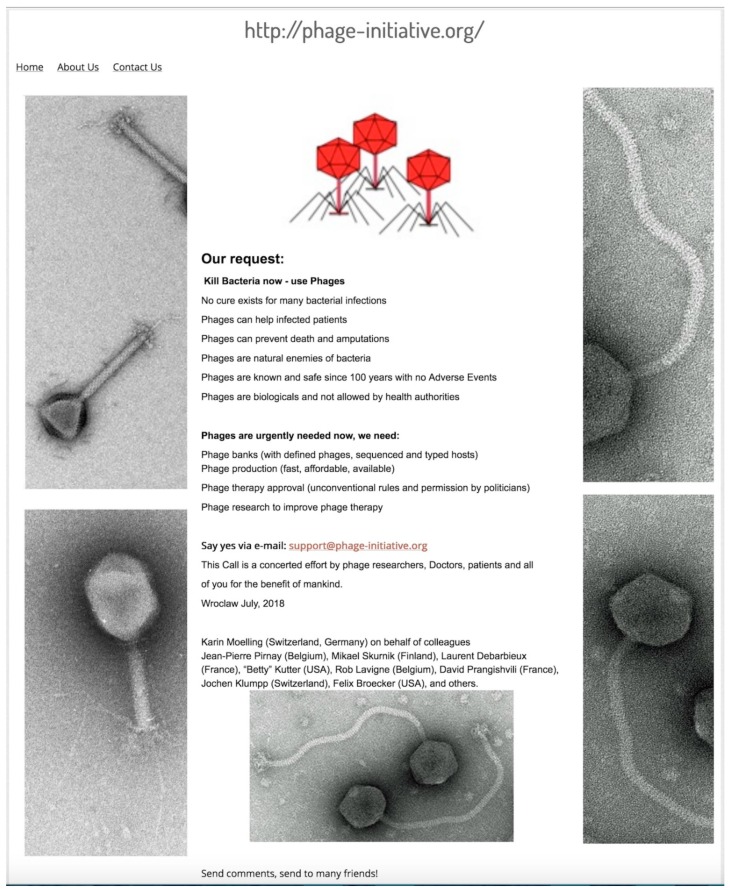
Screenshot of the website http://phage-initiative.org/. Please send an e-mail to: support@phage-initiative.org and say “yes” if you support the initiative.

This proposal was initiated during the Viruses of Microbes workshop in Wroclaw, 2018, by a consortium of several phage researchers, asking all participants for support of phage therapy. Around two hundred participants, including many phage experts, agreed. The names of the consortium members are listed on the petition page shown below (Figure 4). Additional information can be found on www.phage-initiative.org, including references about ongoing research under “About Us”. To support the proposal, please send an e-mail saying “yes” to support@phage-initiative.org.

Many supporting signatures arrived. Almost all participants of the phage meeting signed, but more signatures will be required to convince political authorities. They will be approached with the list of signatures.

We should remember that a global effort was made to successfully combat HIV/AIDS. Similar mechanisms are required to help to proceed with phage therapy. The need is of similar urgency, the number of people suffering from multidrug-resistant infections will be much higher in the near future.

## References

[1] Pirnay J.P., Verbeken G., Ceyssens P.J., Huys I., de Vos D., Ameloot C., Fauconnier A. (2018). The Magistral Phage. Viruses.

[2] Lin D.M., Koskella B., Lin H.C. (2017). Phage therapy: An alternative to antibiotics in the age of multi-drug resistance. World J. Gastrointest. Pharmacol. Ther..

[3] (2013). Press Briefing Transcript: CDC Telebriefing on Today’s Drug-Resistant Health Threats. https://www.cdc.gov/media/releases/2013/t0916_health-threats.html.

[4] Rice L.B. (2008). Federal funding for the study of antimicrobial resistance in nosocomial pathogens: No ESKAPE. J. Infect. Dis..

[5] Comeau A.M., Hatfull G.F., Krisch H.M., Lindell D., Mann N.H., Prangishvili D. (2008). Exploring the prokaryotic virosphere. Res. Microbiol..

[6] Weinbauer M.G. (2004). Ecology of prokaryotic viruses. FEMS Microbiol. Rev..

[7] Suttle C.A. (2005). Viruses in the sea. Nature.

[8] Suttle C.A. (2013). Viruses: Unlocking the greatest biodiversity on Earth. Genome.

[9] Bergh O., Børsheim K.Y., Bratbak G., Heldal M. (1989). High abundance of viruses found in aquatic environments. Nature.

[10] Brum J.R., Ignacio-Espinoza J.C., Roux S., Doulcier G., Acinas S.G., Alberti A., Chaffron S., Cruaud C., de Vargas C., Gasol J.M. (2015). Ocean plankton. Patterns and ecological drivers of ocean viral communities. Science.

[11] Breitbart M., Bonnain C., Malki K., Sawaya N.A. (2018). Phage puppet masters of the marine microbial realm. Nat. Microbiol..

[12] Dublanchet A., Bourne S. (2007). The epic of phage therapy. Can. J. Infect. Dis. Med. Microbiol..

[13] Twort F.W. (1915). An Investigation on the Nature of Ultra-Microscopic Viruses. Lancet.

[14] Summers W.C. (2001). Bacteriophage therapy. Annu. Rev. Microbiol..

[15] Hankin E.H. (1896). L’action bactericide des eaux de la Jumna et du Gange sur le vibrion du cholera (in French). Ann. Inst. Pasteur.

[16] Abedon S.T., Thomas-Abedon C., Thomas A., Mazure H. (2011). Bacteriophage prehistory: Is or is not Hankin, 1896, a phage reference?. Bacteriophage.

[17] Anisimov A.P., Amoako K.K. (2006). Treatment of plague: Promising alternatives to antibiotics. J. Med. Microbiol..

[18] (1911). Cow-dung floors and plague prevention. Lancet.

[19] Lewin R.A. (2001). More on Merde. Perspect. Biol. Med..

[20] Hoffmann-Krayer E., Bächtold-Stäubli H. (1974). Handwörterbuch des Deutschen Aberglaubens (in German).

[21] Manrique P., Dills M., Young M.J. (2017). The Human Gut Phage Community and Its Implications for Health and Disease. Viruses.

[22] D’Herelle F. (1931). Bacteriophage as a Treatment in Acute Medical and Surgical Infections. Bull. N. Y. Acad. Med..

[23] Kutter E., de Vos D., Gvasalia G., Alavidze Z., Gogokhia L., Kuhl S., Abedon S.T. (2010). Phage therapy in clinical practice: Treatment of human infections. Curr. Pharm. Biotechnol..

[24] Tsulukidze A.P. (1941). Experience of Use of Bacteriophages in the Conditions of War Traumatism (in Russian).

[25] Villaroel J., Larsen M.V., Kilstrup M., Nielsen M. (2017). Metagenomic Analysis of Therapeutic PYO Phage Cocktails from 1997 to 2014. Viruses.

[26] Häusler T. (2006). Viruses vs. Superbugs: A Solution to the Antibiotics Crisis?.

[27] Quinn R. (2013). Rethinking antibiotic research and development: World War II and the penicillin collaborative. Am. J. Public Health..

[28] Dublanchet A., Fruciano E. (2008). Brève histoire de la phagothérapie (in French). Med. Mal. Infect..

[29] Broecker F., Kube M., Klumpp J., Schuppler M., Biedermann L., Hecht J., Hombach M., Keller P.M., Rogler G., Moelling K. (2013). Analysis of the intestinal microbiome of a recovered Clostridium difficile patient after fecal transplantation. Digestion.

[30] Broecker F., Russo G., Klumpp J., Moelling K. (2017). Stable core virome despite variable microbiome after fecal transfer. Gut Microbes.

[31] Broecker F., Klumpp J., Schuppler M., Russo G., Biedermann L., Hombach M., Rogler G., Moelling K. (2016). Long-term changes of bacterial and viral compositions in the instestine of a recovered Clostridium difficile patient after fecal microbiota transplantation. Cold Spring Harb. Mol. Case Stud..

[32] Reyes A., Semenkovich N.P., Whiteson K., Rohwer F., Gordon J.I. (2012). Going viral: Next-generation sequencing applied to phage populations in the human gut. Nat. Rev. Microbiol..

[33] Dalmasso M., Hill C., Ross R.P. (2014). Exploiting gut bacteriophages for human health. Trends Microbiol..

[34] Chehoud C., Dryga A., Hwang Y., Nagy-Szakal D., Hollister E.B., Luna R.A., Versalovic J., Kellermayer R., Bushman F.D. (2016). Transfer of Viral Communities between Human Individuals during Fecal Microbiota Transplantation. MBio.

[35] (2014). Critical views in gastroenterology & hepatology: Fecal microbiota transplantation: Where is it leading?. Gastroenterol. Hepatol..

[36] Zhang F., Luo W., Shi Y., Fan Z., Ji G. (2012). Should we standardize the 1,700-year-old fecal microbiota transplantation?. Am. J. Gastroenterol..

[37] Moelling K., Broecker F. (2016). Fecal Microbiota Transplantation to Fight Clostridium difficile Infections and other Intestinal Diseases. Bacteriophage.

[38] Broecker F., Klumpp J., Moelling K. (2016). Long-term microbiota and virome in a Zürich patient after fecal transplantation against Clostridium difficile infection. Ann. N. Y. Acad. Sci..

[39] Filip M., Tzaneva V., Dumitrascu D.L. (2018). Fecal transplantation: Digestive and extradigestive clinical applications. Clujul Med..

[40] Yadav H., Jain S., Nagpal R., Marotta F. (2016). Increased fecal viral content associated with obesity in mice. World J. Diabetes.

[41] Kim M.S., Bae J.W. (2016). Spatial disturbances in altered mucosal and luminal gut viromes of diet-induced obese mice. Environ. Microbiol..

[42] Lepage P., Colombet J., Marteau P., Sime-Ngando T., Doré J., Leclerc M. (2008). Dysbiosis in inflammatory bowel disease: A role for bacteriophages?. Gut.

[43] Norman J.M., Handley S.A., Baldridge M.T., Droit L., Liu C.Y., Keller B.C., Kambal A., Monaco C.L., Zhao G., Fleshner P. (2015). Disease-specific alterations in the enteric virome in inflammatory bowel disease. Cell.

[44] Moelling K., Broecker F., Russo G., Sunagawa S. (2017). RNase H As Gene Modifier, Driver of Evolution and Antiviral Defense. Front. Microbiol..

[45] Ott S.J., Waetzig G.H., Rehman A., Moltzau-Anderson J., Bharti R., Grasis J.A., Cassidy L., Tholey A., Fickenscher H., Seegert D. (2017). Efficacy of Sterile Fecal Filtrate Transfer for Treating Patients with Clostridium Difficile Infection. Gastroenterology.

[46] Zuo T., Wong S.H., Lam K., Lui R., Cheung K., Tang W., Ching J.Y.L., Chan P.K.S., Chan M.C.W., Wu J.C.Y. (2018). Bacteriophage transfer during faecal microbiota transplantation in Clostridium difficile infection is associated with treatment outcome. Gut.

[47] Petrof E.O., Gloor G.B., Vanner S.J., Weese S.J., Carter D., Daigneault M.C., Brown E.M., Schroeter K., Allen-Vercoe E. (2013). Stool substitute transplant therapy for the eradication of Clostridium difficile infection: ’RePOOPulating’ the gut. Microbiome.

[48] Nale J.Y., Redgwell T.A., Millard A., Clokie M.R.J. (2018). Efficacy of an Optimised Bacteriophage Cocktail to Clear Clostridium difficile in a Batch Fermentation Model. Antibiotics.

[49] Ma Y., You X., Mai G., Tokuyasu T., Liu C. (2018). A human gut phage catalog correlates the gut phageome with type 2 diabetes. Microbiome.

[50] Barr J.J., Auro R., Furlan M., Whiteson K.L., Erb M.L., Pogliano J., Stotland A., Wolkowicz R., Cutting A.S., Doran K.S. (2013). Bacteriophage adhering to mucus provide a non-host-derived immunity. Proc. Natl. Acad. Sci. USA.

[51] Nguyen S., Baker K., Padman B.S., Patwa R., Dunstan R.A., Weston T.A., Schlosser K., Bailey B., Lithgow T., Lazarou M. (2017). Bacteriophage Transcytosis Provides a Mechanism to Cross Epithelial Cell Layers. MBio.

[52] Ridaura V.K., Faith J.J., Rey F.E., Cheng J., Duncan A.E., Kau A.L., Griffin N.W., Lombard V., Henrissat B., Bain J.R. (2013). Gut microbiota from twins discordant for obesity modulate metabolism in mice. Science.

[53] Bäckhed F., Ding H., Wang T., Hooper L.V., Koh G.Y., Nagy A., Semenkovich C.F., Gordon J.I. (2004). The gut microbiota as an environmental factor that regulates fat storage. Proc. Natl. Acad. Sci. USA.

[54] Ley R.E., Bäckhed F., Turnbaugh P., Lozupone C.A., Knight R.D., Gordon J.I. (2005). Obesity alters gut microbial ecology. Proc. Natl. Acad. Sci. USA.

[55] Turnbaugh P.J., Ley R.E., Mahowald M.A., Magrini V., Mardis E.R., Gordon J.I. (2006). An obesity-associated gut microbiome with increased capacity for energy harvest. Nature.

[56] Le Chatelier E., Nielsen T., Qin J., Prifti E., Hildebrand F., Falony G., Almeida M., Arumugam M., Batto J.M., Kennedy S. (2013). Richness of human gut microbiome correlates with metabolic markers. Nature.

[57] Menni C., Jackson M.A., Pallister T., Steves C.J., Spector T.D., Valdes A.M. (2017). Gut microbiome diversity and high-fibre intake are related to lower long-term weight gain. Int. J. Obes..

[58] Sulakvelidze A., Alavidze Z., Morris J.G. (2001). Bacteriophage therapy. Antimicrob. Agents Chemother..

[59] Kutateladze M., Adamia R. (2010). Bacteriophages as potential new therapeutics to replace or supplement antibiotics. Trends Biotechnol..

[60] Sabitova Y., Fomenko N., Tikunov A., Stronin O., Khasnatinov M., Abmed D., Danchinova G., Golovljova I., Tikunova N. (2018). Multilocus sequence analysis of Borrelia burgdorferi sensu lato isolates from Western Siberia, Russia and Northern Mongolia. Infect. Genet. Evol..

[61] Morozova V., Kozlova Y., Shedko E., Babkin I., Kurilshikov A., Bokovaya O., Bardashova A., Yunusova A., Tikunov A., Tupikin A. (2018). Isolation and characterization of a group of new Proteus bacteriophages. Arch. Virol..

[62] Górski A., Miedzybrodzki R., Borysowski J., Weber-Dabrowska B., Lobocka M., Fortuna W., Letkiewicz S., Zimecki M., Filby G. (2009). Bacteriophage therapy for the treatment of infections. Curr. Opin. Investig. Drugs.

[63] Wright A., Hawkins C.H., Anggård E.E., Harper D.R. (2009). A controlled clinical trial of a therapeutic bacteriophage preparation in chronic otitis due to antibiotic-resistant Pseudomonas aeruginosa; a preliminary report of efficacy. Clin. Otolaryngol..

[64] Schooley R.T., Biswas B., Gill J.J., Hernandez-Morales A., Lancaster J., Lessor L., Barr J.J., Reed S.L., Rohwer F., Benler S. (2017). Development and Use of Personalized Bacteriophage-Based Therapeutic Cocktails to Treat a Patient with a Disseminated Resistant Acinetobacter baumannii Infection. Antimicrob. Agents Chemother..

[65] Borrud G. German Hospital Gripped by Outbreak of Multiresistant Bacteria. https://p.dw.com/p/1EQZB.

[66] Dettori M., Piana A., Deriu M.G., Lo Curto P., Cossu A., Musumeci R., Cocuzza C., Astone V., Contu M.A., Sotgiu G. (2014). Outbreak of multidrug-resistant Acinetobacter baumannii in an intensive care unit. New Microbiol..

[67] Merino M., Poza M., Roca I., Barba M.J., Sousa M.D., Vila J., Bou G. (2014). Nosocomial outbreak of a multiresistant Acinetobacter baumannii expressing OXA-23 carbapenemase in Spain. Microb. Drug Resist..

[68] Kohlenberg A., Brümmer S., Higgins P.G., Sohr D., Piening B.C., de Grahl C., Halle E., Rüden H., Seifert H. (2009). Outbreak of carbapenem-resistant Acinetobacter baumannii carrying the carbapenemase OXA-23 in a German university medical centre. J. Med. Microbiol..

[69] Molter G., Seifert H., Mandraka F., Kasper G., Weidmann B., Hornei B., Öhler M., Schwimmbeck P., Kröschel P., Higgins P.G., Reuter S. (2016). Outbreak of carbapenem-resistant Acinetobacter baumannii in the intensive care unit: A multi-level strategic management approach. J. Hosp. Infect..

[70] Hujer A.M., Higgins P.G., Rudin S.D., Buser G.L., Marshall S.H., Xanthopoulou K., Seifert H., Rojas L.J., Domitrovic T.N., Cassidy P.M. (2017). Nosocomial Outbreak of Extensively Drug-Resistant Acinetobacter baumannii Isolates Containing bla(OXA-237) Carried on a Plasmid. Antimicrob. Agents Chemother..

[71] Sarker S.A., Berger B., Deng Y., Kieser S., Foata F., Moine D., Descombes P., Sultana S., Huq S., Bardhan P.K. (2017). Oral application of Escherichia coli bacteriophage: Safety tests in healthy and diarrheal children from Bangladesh. Environ. Microbiol..

[72] Kieser S., Sarker S.A., Sakwinska O., Foata F., Sultana S., Khan Z., Islam S., Porta N., Combremont S., Betrisey B. (2018). Bangladeshi children with acute diarrhea show fecal microbiomes with increased Streptococcus abundance, irrespective of diarrhea etiology. Environ. Microbiol..

[73] Jault P., Leclerc T., Jennes S., Pirnay J.P., Que Y.A., Resch G., Rousseau A.F., Ravat F., Carsin H., Le Floch R. (2018). Efficacy and tolerability of a cocktail of bacteriophages to treat burn wounds infected by Pseudomonas aeruginosa (PhagoBurn): A randomised, controlled, double-blind phase 1/2 trial. Lancet Infect. Dis..

[74] Vogt D., Sperling S., Tkhilaishvili T., Trampuz A., Pirnay J.P., Willy C. (2017). “Beyond antibiotic therapy”—Zukünftige antiinfektiöse Strategien—Update 2017 (in German). Unfallchirurg.

[75] Oechslin F., Piccardi P., Mancini S., Gabard J., Moreillon P., Entenza J.M., Resch G., Que Y.A. (2017). Synergistic interaction between phage therapy and antibiotics clears Pseudomonas aeruginosa infection in endocarditis and reduces virulence. J. Infect. Dis..

[76] Valério N., Oliveira C., Jesus V., Branco T., Pereira C., Moreirinha C., Almeida A. (2017). Effects of single and combined use of bacteriophages and antibiotics to inactivate Escherichia coli. Virus Res..

[77] Ryan E.M., Alkawareek M.Y., Donnelly R.F., Gilmore B.F. (2012). Synergistic phage-antibiotic combinations for the control of Escherichia coli biofilms in vitro. FEMS Immunol. Med. Microbiol..

[78] Chaudhry W.N., Concepción-Acevedo J., Park T., Andleeb S., Bull J.J., Levin B.R. (2017). Synergy and order effects of antibiotics and phages in killing Pseudomonas aeruginosa biofilms. PLoS ONE.

[79] Fish R., Kutter E., Wheat G., Blasdel B., Kutateladze M., Kuhl S. (2016). Bacteriophage treatment of intransigent diabetic toe ulcers: A case series. J. Wound Care.

[80] Taha O.A., Connerton P.L., Connerton I.F., El-Shibiny A. (2018). Bacteriophage ZCKP1: A potential treatment for Klebsiella pneumoniae isolated from diabetic foot patients. Front. Microbiol..

[81] Centers for Disease Control and Prevention (2017). National Diabetes Statistics Report. https://www.cdc.gov/diabetes/pdfs/data/statistics/national-diabetes-statistics-report.pdf.

[82] Sybesma W., Zbinden R., Chanishvili N., Kutateladze M., Chkhotua A., Ujmajuridze A., Mehnert U., Kessler T.M. (2016). Bacteriophages as Potential Treatment for Urinary Tract Infections. Front. Microbiol..

[83] Leitner L., Sybesma W., Chanishvili N., Goderdzishvili M., Chkhotua A., Ujmajuridze A., Schneider M.P., Sartori A., Mehnert U., Bachmann L.M., Kessler T.M. (2017). Bacteriophages for treating urinary tract infections in patients undergoing transurethral resection of the prostate: A randomized, placebo-controlled, double-blind clinical trial. BMC Urol..

[84] Ujmajuridze A., Chanishvili N., Goderdzishvili M., Leitner L., Mehnert U., Chkhotua A., Kessler T.M., Sybesma W. (2018). Adapted Bacteriophages for Treating Urinary Tract Infections. Front. Microbiol..

[85] World Medical Association (2013). World Medical Association Declaration of Helsinki: Ethical principles for medical research involving human subjects. JAMA.

[86] Loc-Carrillo C., Abedon S.T. (2011). Pros and cons of phage therapy. Bacteriophage.

[87] Skurnik M., Strauch E. (2006). Phage therapy: Facts and fiction. Int. J. Microbiol..

[88] Ormälä A.M., Jalasvuori M. (2013). Phage therapy: Should bacterial resistance to phages be a concern, even in the long run?. Bacteriophage.

[89] Mölling K. (2017). Viren statt Antibiotika (in German). Spektrum der Wissenschaft.

[90] Reindel R., Fiore C.R. (2017). Phage Therapy: Considerations and Challenges for Development. Clin. Infect. Dis..

[91] Hagens S., Habel A., von Ahsen U., von Gabain A., Bläsi U. (2004). Therapy of experimental pseudomonas infections with a nonreplicating genetically modified phage. Antimicrob. Agents Chemother..

[92] Dufour N., Delattre R., Ricard J.D., Debarbieux L. (2017). The Lysis of Pathogenic Escherichia coli by Bacteriophages Releases Less Endotoxin Than by β-Lactams. Clin. Infect. Dis..

[93] Fighting AMR: Turning the p(h)age. 22.05.2018. https://european-biotechnology.com/up-to-date/backgrounds-stories/story/fighting-amr-turning-the-phage.html.

[94] Bourdin G., Schmitt B., Marvin Guy L., Germond J.E., Zuber S., Michot L., Reuteler G., Brüssow H. (2014). Amplification and purification of T4-like Escherichia coli phages for phage therapy: From laboratory to pilot scale. Appl. Environ. Microbiol..

[95] Fauconnier A. (2017). Regulating phage therapy: The biological master file concept could help to overcome regulatory challenge of personalized medicines. EMBO Rep..

